# Potential Indirect Effects of the COVID-19 Pandemic on Use of Emergency Departments for Acute Life-Threatening Conditions — United States, January–May 2020

**DOI:** 10.15585/mmwr.mm6925e2

**Published:** 2020-06-26

**Authors:** Samantha J. Lange, Matthew D. Ritchey, Alyson B. Goodman, Taylor Dias, Evelyn Twentyman, Jennifer Fuld, Laura A. Schieve, Giuseppina Imperatore, Stephen R. Benoit, Aaron Kite-Powell, Zachary Stein, Georgina Peacock, Nicole F. Dowling, Peter A. Briss, Karen Hacker, Adi V. Gundlapalli, Quanhe Yang

**Affiliations:** ^1^National Center for Chronic Disease Prevention and Health Promotion, CDC; ^2^CDC COVID-19 Emergency Response; ^3^Center for Surveillance, Epidemiology, and Laboratory Services, CDC.

On March 13, 2020, the United States declared a national emergency in response to the coronavirus disease 2019 (COVID-19) pandemic. Subsequently, states enacted stay-at-home orders to slow the spread of SARS-CoV-2, the virus that causes COVID-19, and reduce the burden on the U.S. health care system. CDC* and the Centers for Medicare & Medicaid Services (CMS)^†^ recommended that health care systems prioritize urgent visits and delay elective care to mitigate the spread of COVID-19 in health care settings. By May 2020, national syndromic surveillance data found that emergency department (ED) visits had declined 42% during the early months of the pandemic (*1*). This report describes trends in ED visits for three acute life-threatening health conditions (myocardial infarction [MI, also known as heart attack], stroke, and hyperglycemic crisis), immediately before and after declaration of the COVID-19 pandemic as a national emergency. These conditions represent acute events that always necessitate immediate emergency care, even during a public health emergency such as the COVID-19 pandemic. In the 10 weeks following the emergency declaration (March 15–May 23, 2020), ED visits declined 23% for MI, 20% for stroke, and 10% for hyperglycemic crisis, compared with the preceding 10-week period (January 5–March 14, 2020). EDs play a critical role in diagnosing and treating life-threatening conditions that might result in serious disability or death. Persons experiencing signs or symptoms of serious illness, such as severe chest pain, sudden or partial loss of motor function, altered mental state, signs of extreme hyperglycemia, or other life-threatening issues, should seek immediate emergency care, regardless of the pandemic. Clear, frequent, highly visible communication from public health and health care professionals is needed to reinforce the importance of timely care for medical emergencies and to assure the public that EDs are implementing infection prevention and control guidelines that help ensure the safety of their patients and health care personnel.

CDC used data from its National Syndromic Surveillance Program (NSSP) to assess trends in ED visits from week 1, 2019 through week 21, 2020 for three life-threatening health conditions: MI, stroke, and hyperglycemic crisis. NSSP is a collaboration among CDC, federal partners, local and state health departments, and academic and private sector partners to collect, analyze, and share electronic patient encounter data received from emergency departments, urgent and ambulatory care centers, inpatient health care settings, and laboratories for public health action.^§^ NSSP includes ED visits from a subset of hospitals in 47 states (all but Hawaii, South Dakota, and Wyoming) and the District of Columbia, capturing approximately 73% of ED visits nationwide. These analyses were limited to EDs with consistent ≥90% completeness for patient discharge diagnosis to ensure data quality (1,670 EDs).^¶^ The three conditions were defined using the following *International Classification of Diseases, Tenth Revision* (ICD-10) codes: MI = I21–I22; stroke = I60–I61 (hemorrhagic stroke) or I63 (ischemic stroke); and hyperglycemic crisis = E10.1, E11.1, or E13.1 (diabetic ketoacidosis) or E11.0, E13.0, or E10.65 and E10.69 (hyperosmolar hyperglycemic syndrome). Weekly numbers of ED visits for each of the three conditions were compared for two 10-week periods: January 5–March 14, 2020 (weeks 2–11, prepandemic) and March 15–May 23, 2020 (weeks 12–21, early pandemic). The absolute differences and percentage change in number of visits from pre- to early pandemic periods were tabulated, overall and within age-sex strata. Analyses were conducted using SAS (version 9.4; SAS Institute).

Trends in number of ED visits for MI and stroke were relatively stable during the first half of 2019, increased slightly in the second half of 2019, and then stabilized during the first few weeks of 2020, remaining stable throughout the prepandemic period (Figure 1). The number of ED visits for MI and stroke declined sharply starting at week 10 (corresponding to the week beginning March 1, 2020) and reaching the lowest level during weeks 13–14 (weeks beginning March 22 for MI and March 29 for stroke), coinciding with the early weeks after the declaration of the COVID-19 national emergency. Since the nadir, ED visits for MI and stroke have gradually increased but remain below prepandemic levels. Compared with the prepandemic period, the number of ED visits during the early pandemic period was 23% lower for MI and 20% lower for stroke (Table). The number of ED visits for hyperglycemic crisis followed similar, albeit less pronounced, trends to those observed for MI and stroke; the number of ED visits for hyperglycemic crisis was 10% lower during the early pandemic than during the prepandemic period, with the lowest level occurring at week 14. The reduction in visits for all three conditions during the early pandemic was similar in males and females.

**FIGURE 1:**
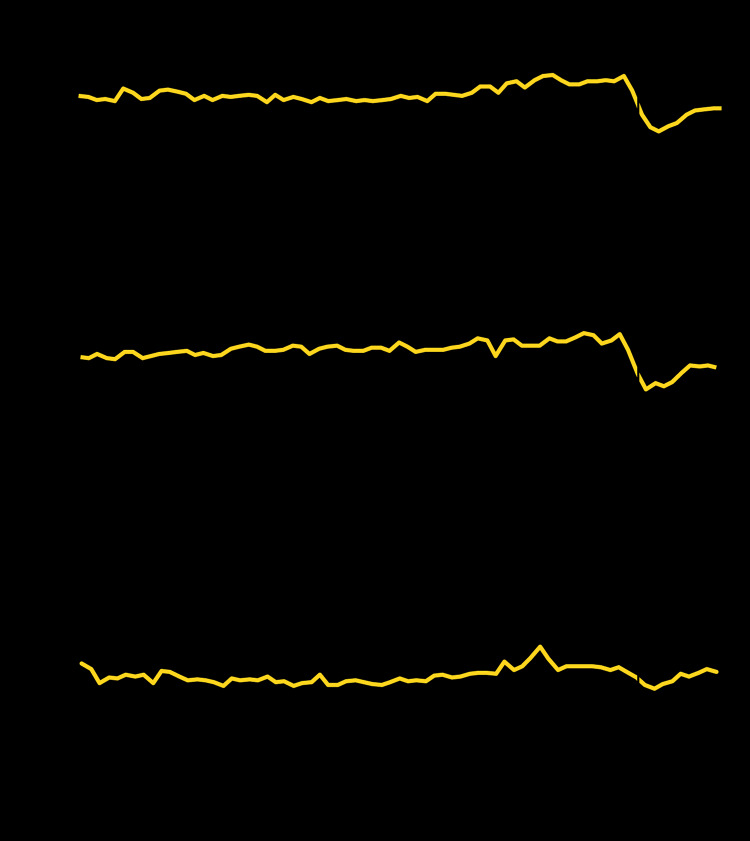
Number of emergency department (ED) visits for myocardial infarction, stroke, and hyperglycemic crisis* — National Syndromic Surveillance Program, United States, week 1, 2019–week 21, 2020^†^ **Abbreviation:** COVID-19 = coronavirus disease 2019. * Includes diabetic ketoacidosis and hyperosmolar hyperglycemic syndrome. ^†^ Week 1, 2019 (week ending January 5, 2019) to week 21, 2020 (week ending May 23, 2020).

**TABLE:** Number of emergency department visits and percentage change for myocardial infarction, stroke, and hyperglycemic crisis immediately before and during the early COVID-19 pandemic, by sex and age group — National Syndromic Surveillance Program, United States, 2020

Sex/Age	Myocardial infarction			Stroke			Hyperglycemic crisis		
	Prepandemic*	Early pandemic^†^	% Change	Prepandemic	Early pandemic	% Change	Prepandemic	Early pandemic	% Change
**Total**	**56,565**	**43,545**	**−23**	**57,490**	**46,066**	**−20**	**22,766**	**20,561**	**−10**
**Males**	33,263	26,176	−21	28,729	23,715	−17	11,842	11,070	−7
**Age group (yrs)**									
<18	10	5	−50	169	180	7	895	779	−13
18–44	2,101	1,805	−14	1,984	1,765	−11	5,236	4,817	−8
45–54	4,510	3,669	−19	3,256	2,665	−18	2,025	1,958	−3
55–64	8,228	6,780	−18	6,488	5,518	−15	1,887	1,854	−2
65–74	8,965	6,851	−24	7,532	6,126	−19	1,120	1,042	−7
75–84	6,218	4,736	−24	6,083	4,998	−18	526	490	−7
≥85	3,231	2,330	−28	3,217	2,463	−23	153	130	−15
**Females**	23,017	17,128	−26	28,666	22,260	−22	10,888	9,469	−13
**Age group (yrs)**									
<18	8	0	−100	137	100	−27	902	685	−24
18–44	1,168	882	−24	1,787	1,428	−20	4,775	4,000	−16
45–54	2,131	1,632	−23	2,625	2,050	−22	1,613	1,503	−7
55–64	4,396	3,372	−23	4,683	3,850	−18	1,689	1,509	−11
65–74	5,782	4,323	−25	6,625	5,056	−24	1,173	1,038	−12
75–84	5,379	3,924	−27	7,006	5,364	−23	536	540	1
≥85	4,153	2,995	−28	5,803	4,412	−24	200	194	−3
**Sex unknown**	285	241	−15	95	91	−4	36	22	−39

**Abbreviation:** COVID-19 = coronavirus disease 2019.

* Prepandemic (weeks 2–11) corresponds to January 5–March 14, 2020.

^†^ Early pandemic (weeks 12–21) corresponds to March 15–May 23, 2020.

The relative decline in the number of ED visits between the prepandemic and early pandemic periods was similar across age groups for MI and stroke, whereas the decline in ED visits for hyperglycemic crisis tended to be larger among younger age groups, particularly for females (Table). The absolute decrease in ED visits for MI was largest among persons aged 65–74 years for both men (2,114-visit decrease) and women (1,459) (Figure 2). The absolute decrease in ED visits for stroke was largest among men aged 65–74 years (1,406-visit decrease) and women aged 75–84 years (1,642). The absolute decrease in ED visits for hyperglycemic crisis was largest in younger adults aged 18–44 years (419-visit decrease for men, 775 for women).

**FIGURE 2:**
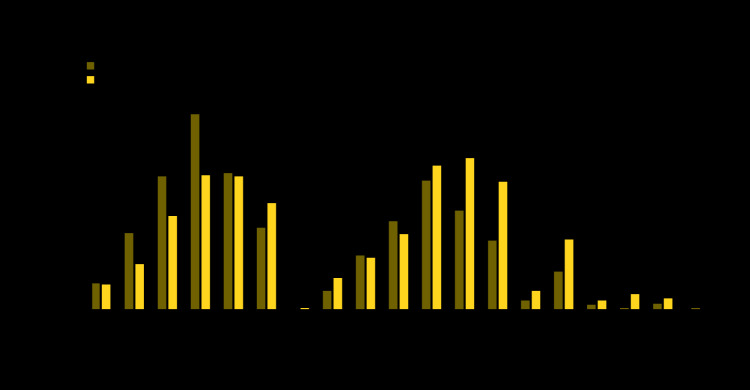
Absolute decreases in number of emergency department (ED) visits for myocardial infarction, stroke, and hyperglycemic crisis between COVID-19 prepandemic* and early pandemic periods,^†^ by sex and age group^§^ — National Syndromic Surveillance Program, United States, 2020 **Abbreviation:** COVID-19 = coronavirus disease 2019. * Prepandemic (weeks 2–11) corresponds to January 5–March 14, 2020. ^†^ Early pandemic (weeks 12–21) corresponds to March 15–May 23, 2020. ^§ ^There was a slight absolute increase in ED visits for stroke among males aged 0–17 years and for hyperglycemic crisis among females aged 75–84 years.

## Discussion

In the weeks following the declaration of COVID-19 as a national emergency on March 13, 2020, NSSP identified substantial reductions in numbers of ED visits by males and females in all age groups for three potentially life-threatening conditions: MI (23% decrease), stroke (20%), and hyperglycemic crisis (10%). These estimates are consistent with, but smaller in relative magnitude than, the 42% overall decline in ED visits observed during the early pandemic period (*1*). The largest absolute differences were observed in adults aged ≥65 years for MI and stroke, and adults aged 18–44 years and persons aged <18 years for hyperglycemic crisis. The substantial reduction in ED visits for these life-threatening conditions might be explained by many pandemic-related factors including fear of exposure to COVID-19, unintended consequences of public health recommendations to minimize nonurgent health care, stay-at-home orders, or other reasons. A short-term decline of this magnitude in the incidence of these conditions is biologically implausible for MI and stroke, especially for older adults, and unlikely for hyperglycemic crisis, and the finding suggests that patients with these conditions either could not access care or were delaying or avoiding seeking care during the early pandemic period. There have been reports of excess mortality during the COVID-19 pandemic wherein deaths not associated with confirmed or probable COVID-19 might have been directly or indirectly attributed to the pandemic.** The striking decline in ED visits for acute life-threatening conditions might partially explain observed excess mortality not associated with COVID-19.

Previous studies have also reported significant reductions in hospital admissions for MI and stroke during the COVID-19 pandemic (*2*–*7*). For example, a study of nine high-volume U.S. cardiac catheterization laboratories found a 38% decrease in activations for heart attacks during March 2020 compared with the 14 months before the pandemic (*2*). Further, large hospital systems in California, Massachusetts, and New York City have reported 43%–50% reductions in admissions for MI and other acute cardiovascular conditions during the pandemic (*3*–*5*), and neuroimaging data from approximately 850 U.S. hospitals indicate a 39% reduction in the number of patients who were evaluated for signs of stroke (*7*). Decreases in ED visits for hyperglycemic crisis might be less striking because patient recognition of this crisis is typically augmented by home glucose monitoring and not reliant upon symptoms alone, as is the case for MI and stroke. The decrease in visits for hyperglycemic crisis merits further study because there are few published reports on this topic.

MI, stroke, and hyperglycemic crisis are common life-threatening conditions that require urgent attention to reduce associated morbidity and mortality. Heart disease is the leading cause of death, and stroke is the fifth leading cause of death in the United States^††^: someone in the United States has a heart attack every 40 seconds,^§§^ and approximately 795,000 persons have a stroke annually.^¶¶^ Diabetes affects 34 million Americans,*** and uncontrolled hyperglycemia (high blood glucose), can lead to diabetic ketoacidosis or a hyperosmolar hyperglycemic state, life-threatening but preventable metabolic complications of diabetes (*8*). It is important for all persons to know the warning signs of MI, stroke, and hyperglycemic crisis^†††^ and understand that immediate medical attention for these acute issues can prevent serious heart or brain damage, metabolic complications of diabetes, or death. The sooner emergency care begins, the better are the chances for survival. Even in the face of the COVID-19 pandemic, emergency care can and should be accessed and provided without delay.

The findings in this report are subject to at least five limitations. First, NSSP coverage is not uniform across or within states, and hospitals reporting to NSSP change over time; however, NSSP captures approximately 73% of the ED data analyzable at the national level. Second, conditions were defined using ICD-10 diagnosis codes. Differences in coding practices might exist; however, coding for common conditions, especially the life-threatening conditions described in this report, is likely consistent (*9*,*10*). Third, NSSP does not capture mortality data, and it is not known whether patients with MI or stroke sought treatment elsewhere or died at home. Fourth, despite allowing 2 weeks from the end of week 21 before analyzing the data, the findings from the final weeks might be slightly underestimated because of delayed reporting. Finally, seasonal effects in trends in ED visits might exist; however, a proximal comparison period was best for this analysis to minimize other factors that might have affected trends in disease incidence or health care–seeking behavior between years. Despite these limitations, this study also has important strengths. NSSP is a national surveillance system with automated electronic reporting and the ability to detect and monitor health events in near real time, and this analysis was restricted to hospitals with consistent reporting on patients’ diagnoses at discharge to minimize effects of differential reporting.

At least one in five expected U.S. ED visits for MI or stroke and one in 10 ED visits for hyperglycemic crisis did not occur during the initial months of the COVID-19 pandemic. Patients might have delayed or avoided seeking care because of fear of COVID-19, unintended consequences of recommendations to stay at home, or other reasons. EDs play a critical role in treating acute conditions that might result in permanent disability or death. Persons experiencing severe chest pain, sudden or partial loss of motor function, altered mental status, signs of extreme hyperglycemia, or other life-threatening issues, should call 9-1-1, irrespective of the COVID-19 pandemic. Clear communication from public health and health care professionals is needed to reinforce the importance of timely emergency care for acute health conditions and to assure the public that EDs are implementing infection prevention and control guidelines^§§§^ to ensure the safety of their patients and health care personnel.

**Summary**

**What is already known about this topic?**

National syndromic surveillance data suggest a decline in emergency department (ED) visits during the COVID-19 pandemic.

**What is added by this report?**

In the 10 weeks following declaration of the COVID-19 national emergency, ED visits declined 23% for heart attack, 20% for stroke, and 10% for hyperglycemic crisis.

**What are the implications for public health practice?**

Persons experiencing chest pain, loss of motor function, altered mental status, or other life-threatening issues should seek immediate emergency care, regardless of the pandemic. Communication from public health and health care professionals should reinforce the importance of timely care for acute health conditions and assure the public that EDs are implementing infection prevention and control guidelines to ensure the safety of patients and health care personnel.

## Footnotes

*
https://www.cdc.gov/coronavirus/2019-ncov/hcp/framework-non-COVID-care.html .

†
https://www.cms.gov/files/document/cms-non-emergent-elective-medical-recommendations.pdf .

§
https://www.cdc.gov/nssp/index.html .

¶ During weeks 2–21, 2020, an average of 3,504 EDs reported to NSSP. On average, 1,670 EDs (48%) had consistent (≥90%) completeness on patient discharge diagnosis data during this period.

** https://www.cdc.gov/mmwr/volumes/69/wr/mm6919e5.htm.

††
https://www.cdc.gov/nchs/fastats/leading-causes-of-death.htm .

§§
https://www.cdc.gov/heartdisease/facts.htm .

¶¶
https://www.cdc.gov/stroke/facts.htm .

***
https://www.cdc.gov/diabetes/pdfs/data/statistics/national-diabetes-statistics-report.pdf .

††† The five major symptoms of MI, or heart attack, are chest pain or discomfort; feeling weak, light-headed, or faint; pain or discomfort in the jaw, neck, or back; pain or discomfort in one or both arms or shoulders; and shortness of breath. The F.A.S.T acronym is a mnemonic that might help determine whether someone is having a stroke: F = Face: When the person smiles, does one side of the face droop? A = Arms: When the person tries to raise both arms, does one arm drift downward? S = Speech: When the person tries to repeat a simple phrase, is the speech slurred or strange? T = Time: If any of these signs are present, persons should call 9–1-1 right away. Signs of hyperglycemic crisis might include low blood pressure, lethargy, dehydration, a confused or altered mental state attributable to high blood glucose in a person with diabetes.

§§§ https://www.cdc.gov/coronavirus/2019-ncov/hcp/infection-control-recommendations.html.
